# Facile and versatile PDMS-glass capillary double emulsion formation device coupled with rapid purification toward microfluidic giant liposome generation

**DOI:** 10.1038/s41378-024-00815-0

**Published:** 2024-12-05

**Authors:** Mostafa Bakouei, Ali Kalantarifard, Indraja Sundara Raju, Tatiana Avsievich, Lauri Rannaste, Marjut Kreivi, Caglar Elbuken

**Affiliations:** 1 Faculty of Biochemistry and Molecular Medicine, University of Oulu, Oulu, Finland; 2 VTT Technical Research Centre of Finland, Oulu, Finland

**Keywords:** Physics, Materials science

## Abstract

The exceptional ability of liposomes to mimic a cellular lipid membrane makes them invaluable tools in biomembrane studies and bottom-up synthetic biology. Microfluidics provides a promising toolkit for creating giant liposomes in a controlled manner. Nevertheless, challenges associated with the microfluidic formation of double emulsions, as precursors to giant liposomes, limit the full exploration of this potential. In this study, we propose a PDMS-glass capillary hybrid device as a facile and versatile tool for the formation of double emulsions which not only eliminates the need for selective surface treatment, a well-known problem with PDMS formation chips, but also provides fabrication simplicity and reusability compared to the glass-capillary formation chips. These advantages make the presented device a versatile tool for forming double emulsions with varying sizes (spanning two orders of magnitude in diameter), shell thickness, number of compartments, and choice of solvents. We achieved robust thin shell double emulsion formation by operating the hybrid chip in double dripping mode without performing hydrophilic/phobic treatment a priori. In addition, as an alternative to the conventional, time-consuming density-based separation method, a tandem separation chip is developed to deliver double emulsions free of any oil droplet contamination in a continuous and rapid manner without any need for operator handling. The applicability of the device was demonstrated by forming giant liposomes using the solvent extraction method. This easy-to-replicate, flexible, and reliable microfluidic platform for the formation and separation of double emulsion templates paves the way for the high-throughput microfluidic generation of giant liposomes and synthetic cells, opening exciting avenues for biomimetic research.

**Figure:**
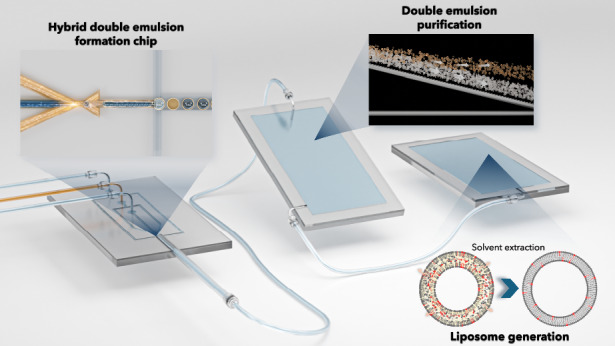
The presented giant liposome assembly line features a novel treatment-free hybrid chip for double emulsion formation coupled with a high throughput separation chip for sample purification.

The presented giant liposome assembly line features a novel treatment-free hybrid chip for double emulsion formation coupled with a high throughput separation chip for sample purification.

## Introduction

Giant liposomes, as models to replicate cell membrane structure, have received growing interest in the field of bottom-up cell biology^1,2^. These structures are instrumental in comprehending living systems and their fundamental building blocks. Microfluidic liposome generation has initiated a new era for engineered synthetic cell generation owing to the high encapsulation efficiency and monodispersity of the output^3–5^. It involves the production of double emulsions (DEs) as templates for liposomes^6,7^. To produce DEs on a chip, the initial step involves the controlled dripping of the inner aqueous phase (IP) by the middle oleaginous phase (MP) forming water in oil (W/O) emulsions. These single emulsions with the carrier oil are then dripped by the outer aqueous phase (OP) forming water in oil in water (W/O/W) double emulsions. Successful droplet formation at each step requires complete wetting of the channel walls by the carrier fluid. Therefore, it is essential to have a hydrophobic surface to generate W/O emulsions and a hydrophilic surface to form DEs within the same chip. However, the need for selective surface treatment in the chip and the challenges associated with obtaining purified liposome samples have limited the use of microfluidic approaches^8–11^. Herein, we address these problems with a microfluidic device with two distinct features: A treatment-free PDMS-glass capillary formation chip and an integrated purification chip.

So far, to form DEs as liposome templates on a microfluidic chip, either a polydimethylsiloxane (PDMS) chip or a glass capillary device has been used, each having its own downsides. For PDMS chips, the major issue is the need for selective hydrophilic surface treatment. Several hydrophilicity treatment techniques have been suggested including polyethylene glycol (PEG)-PDMS surface modification^12^, in-mould modification^13^, polyethylene oxide (PEO)-PDMS coating^14^, polyelectrolyte coating^15^, corona-plasma treatment^11,16^, and polyvinyl alcohol (PVA) treatment^17,18^. However, difficulties involved with these treatment methods render them unreliable and challenging to replicate. Notably, certain treatment methods are implemented for bulk hydrophilicity thus they are unsuitable for selective surface treatment^12,13^. Furthermore, the success of these treatment methods highly relies on meticulous adherence to protocols and requires hands-on expertise^19^. Even for successful treatments, over time the treatment’s efficacy degrades limiting the chip lifetime to minutes/hours. The presence of phospholipids in the middle phase exacerbates the selective treatment problem, as it requires a stronger wettability contrast between lipid and aqueous solutions^20^. Similarly, glass capillary devices^6,21^ not only suffer from hydrophobicity treatment but also from fabrication intricacies, i.e., capillary alignment, sealing of connectors with adhesives that are prone to leaking or clogging and the inability of capillary replacement. Attempts for addressing some of these issues^22–24^, resulted in additional chip fabrication complexity.

In this study, we present our PDMS-glass capillary (hybrid) device as an unparalleled solution, with four unique features: i) treatment-free, ii) reusable, iii) customizable and iv) versatile double emulsion formation. To the best of our knowledge, this is the first demonstration of the PDMS-glass capillary hybrid device structure for liposome generation, where the phospholipids in the middle phase and the need for shell thickness control make DE formation highly challenging^25–28^. The hybrid device integrates the intrinsic hydrophobicity of PDMS and hydrophilicity of glass capillaries, eliminating the need for surface treatment in the formation of W/O/W double emulsions. The hybrid device’s PDMS chip enables the incorporation of tailored designs, e.g., different droplet formation geometries and integration of existing functionalities e.g., mixing and sorting. This customizability is not achievable in glass capillary devices. The glass capillary component of the device ensures prolonged usability and is easy to replace in case of any defect, rendering the chip reusable. The versatility of the device was demonstrated by varying the number of compartments, shell thickness, use of various lipid solvents and different formation modes. To the best of our knowledge, we achieved the most extensive size range of DEs reported thus far, spanning from 27 µm to 1.2 mm.

Another achievement in this study is the development of an on-chip separation method to address a practical issue in the field. To obtain the desired DEs for further investigation, a purification step is required, as the formation could yield both oil droplets and DEs. To do so, a separation chip is proposed in tandem to the hybrid device. This separation method takes advantage of the fluid flow profile and the density difference between the DEs and oil droplets to purify the sample from any oil droplet. Off-chip separation was commonly employed in such cases to isolate the DEs or similarly liposomes from oil droplets^29–32^. The presented on-chip separation method is advantageous due to the removal of additional off-chip processing steps which results in significantly higher DE throughput. It also enables rapid processing, real-time monitoring and is applicable even in scenarios where DEs have lower density than the continuous medium. Finally, to form liposomes from DEs, the solvent extraction method is used^33–35^, demonstrating the capability of our device for giant liposome generation.

## Results and discussion

**Fig. 1:**
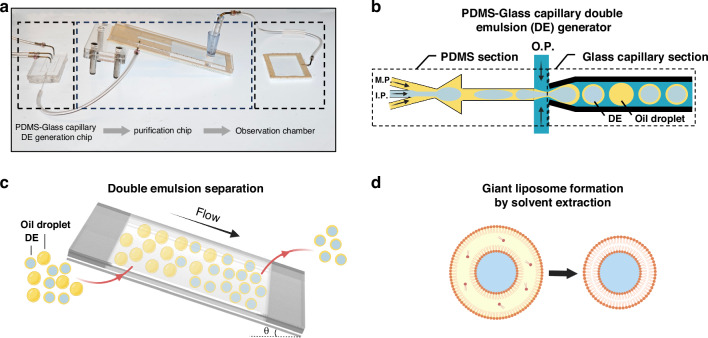
. An illustration of the presented work. **a** The experimental setup for the proposed microfluidic platform consisting of a PDMS-Glass capillary double emulsion generation chip and a tandem purification chip resulting in isolated DE sample in the observation chamber. Schematic representation of (**b**) double emulsion (DE) formation in the PDMS-Glass capillary chip, (**c**) continuous and rapid separation of double emulsions from oil droplets and (**d**) formation of giant liposomes by solvent extraction method as a demonstration of the applicability of presented platform for giant liposome production

As illustrated in Fig. 1, the presented work consists of three sections: first, fabricating the PDMS-Glass capillary (hybrid) microfluidic device for double emulsion formation and demonstrating its versatility; second, purifying the double emulsion sample by a continuous separation method; and third, demonstrating giant liposome formation using the presented platform.

### Hybrid PDMS-glass capillary device a versatile tool for double emulsion formation

**Fig. 2:**
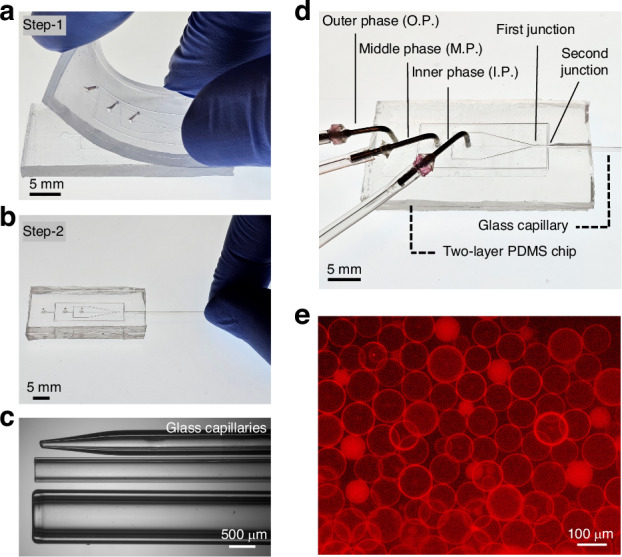
. The hybrid PDMS-glass capillary device. The 2-step assembly illustration of the chip, where (**a**) two identical PDMS replicas are bonded (step 1) and (**b**) a glass capillary is inserted into the outlet channel (step 2). **c** Glass capillaries with different tip ID used for the formation of double emulsions of different sizes. **d** Photograph of the hybrid device which includes a PDMS chip (containing the inlets of three phases and the first junction), and the glass capillary (containing the second junction). The inlet channel heights are 50, 200 and 250 μm respectively for IP, MP, and OP, with square channel cross sections. The glass capillary has 820 μm OD and 550 μm ID. **e** fluorescent image showing the double emulsions formed using the hybrid device

The hybrid device proposed in this study comprises a PDMS microfluidic chip, where the W/O emulsions form, and a glass capillary, where the double emulsion formation takes place. The hybrid chip is assembled in two steps, as depicted in Fig. 2a. The first step involves bonding two identical PDMS replicas, which are cast from a 3D printed mould and subsequently aligned and bonded together to form the PDMS chip. In the second step (Fig. 2b), the glass capillary is seamlessly inserted into the outlet channel of the PDMS chip. The formation of DEs in a broad range of sizes is achieved by inserting capillaries with different tip IDs into the PDMS chip (Fig. 2c). The fabricated hybrid device after the assembly is shown in Fig. 2d. The hydrophobic nature of the PDMS ensures the successful formation of W/O droplets at the first junction. Subsequently, at the second junction, all three phases meet in the glass capillary, where the formation of W/O/W double emulsions occurs. Leveraging the surface properties of the glass capillary eliminates the need for hydrophilicity surface treatment, facilitating the straightforward generation of W/O/W double emulsions. As a result of this combination, DEs have been successfully formed using the hybrid device without any treatment (Fig. 2e). The following sections will explore the versatility and controllability of double emulsion formation using this innovative approach.

#### Size tunability in double emulsion formation

**Fig. 3:**
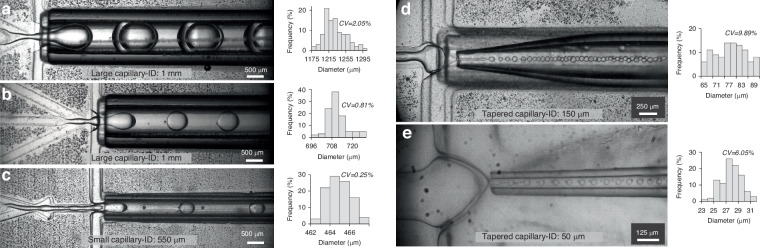
. Size tunability in the formation of thin shell water in oil in water (W/O/W) double emulsions using the hybrid device. **a**, **b** Double emulsions of 1.2 mm and 700 μm diameter generated using the hybrid device designed for the large capillary with 1 mm ID (**c**) Formation of 464 μm double emulsion in the chip equipped for small capillary with 550 μm ID. **d**, **e** The formation of 77 μm and 27 µm thin shell double emulsions using the same chip as (**c**) but replacing the capillary with tapered ones having tip size of 150 μm and 50 μm ID respectively. The size distribution plots and the corresponding coefficient of variation (CV) for the generated DEs are shown on the right side of each figure

Two distinct chips were designed for two sizes of glass capillary (1 mm and 550 μm ID) to investigate the tunability of double emulsion size. Each design was tailored to accommodate the specific dimensions of the capillary. We demonstrate the formation of large double emulsions (DEs) with 1.2 mm (Fig. 3a) and 700 μm (Fig. 3b) diameter using the large capillary (1 mm ID). While it was feasible to form double emulsions as small as 200 μm using the same capillary, the formation frequency and monodispersity of DEs were low. Hence, a capillary with an ID of 550 μm was used for smaller DE formation, as depicted in Fig. 3c. to further enhance size tunability, the same capillary was tapered to have a 150 μm and 50 μm tip ID (Fig. 3d, e), resulting in the formation of thin shell double emulsions as small as 27 μm diameter. These results demonstrate the versatility of the treatment-free hybrid device, where two different designs and interchangeable capillaries with varying tip sizes produce single-compartment thin shell double emulsions ranging from 1.2 mm to 27 μm in size (Video S1†). The DEs formed display monodispersity with a coefficient of variation (CV) in diameter ranging between 0.25 to 10% (Fig. 3) and a formation throughput of up to 1140 DE/s. (See supporting information Fig. S1† and Table S1† for the detailed information.)

#### Controlling the shell thickness of double emulsions

The ability to adjust the double emulsion’s shell thickness enables the hybrid device to be used in generating microcarriers, core-shell microspheres, and synthetic cells^34,36–38^.

**Fig. 4:**
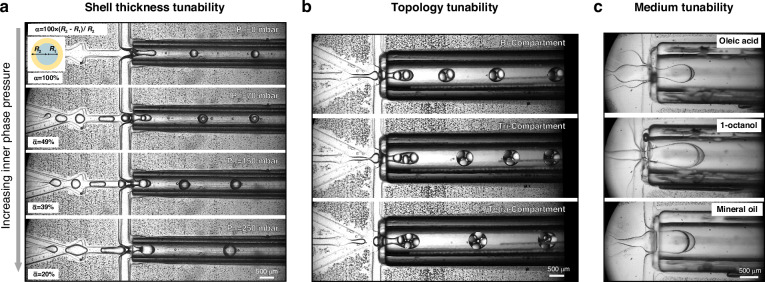
. Tunability of the presented PDMS-Glass capillary device. **a** Controlling the shell thickness of double emulsions by the inner phase pressure (P_IP_). Higher pressure in the IP yields to encapsulation of a larger inner droplet. Hence double emulsions with lower relative shell thickness can be formed. The percentage of the ratio of middle phase thickness (*R*_2_-*R*_1_) to the radius of double emulsion (*R*_2_) is defined as relative shell thickness (α). The MP pressure was set to 60 mbar and OP pressure set to 240 mbar. The same chip design and capillary dimensions explained in Fig. 3c were used for these experiments. Shell thickness uniformity plots and enlarged images of individual DEs are shown in Fig. S2. **b** The formation of bi, tri and tetra-compartment double emulsions. Setting the outer phase pressure to 600 mbar yielded to bi-compartment double emulsions, then by reducing the pressure to about 450 mbar tri-compartment ones were formed. Eventually using 250 mbar results in the formation of tetra-compartment ones. The IP and MP pressure were set to 40 and 50 mbar respectively. **c** The formation of thin shell double emulsions with different middle phase (oleic acid, 1-octanol and mineral oil) using hybrid device. In **b** and **c** chip design and capillary dimensions were the same as in Fig. 3a

The modulation of the DE’s shell thickness in the hybrid device is achieved through the manipulation of inner phase pressure (P_IP_). Figure 4a illustrates the reduction in the relative shell thickness of double emulsion (α = 100 × (*R*_2_−*R*_1_) / *R*_2_) as P_IP_ increases from 0 mbar, forming single emulsions (α = 100%) to 250 mbar, forming thin shell DEs ($\bar{{\rm{\alpha }}}=20{\text{\%}}$). The shell thickness uniformity of the resulting DEs spans between 1.2 to 4.8% CV (Fig. S2†). The formation of ultra-thin shell DE by the hybrid device is demonstrated in the Supplementary Video S2†.

#### Formation of multicompartment double emulsions

The applicability of our hybrid device to form multicompartment double emulsions (DEs) is shown by generating DEs containing 2 to 4 aqueous cores (Fig. 4b). Such structures are used as microcapsules and hydrogel particles^39–41^. Production of these emulsions includes the initial formation of core water in oil (W/O) droplets at the first junction followed by encapsulation within the outer phase leading to W/O/W double emulsions. Depending on the number of encapsulated W/O droplets, DEs are classified into bi, tri or tetra compartment morphologies. In the formation of these multicore DEs, prioritizing a thin shell results in structures with a lower oil content compared to previous studies^11,16^. Such structures are advantageous to obtain multicompartment liposomes or polymersomes^31,42–44^.

#### Formation of double emulsions with different lipid solvents

To highlight the hybrid device’s flexibility, we explored its compatibility with lipid solvents by employing three different oils as the middle phase. We investigated mineral oil, a frequently utilized oil in liposome formation via the inverted emulsion method^45^, along with oleic acid, used for solvent extraction liposomes^33^, and 1-octanol used for octanol-assisted liposomes^46^. As depicted in Fig. 4c, the successful formation of thin shell double emulsions was achieved using these solvents, showcasing the device’s compatibility with the most commonly used lipid solvents.

#### Thin shell double emulsion formation modes

**Fig. 5:**
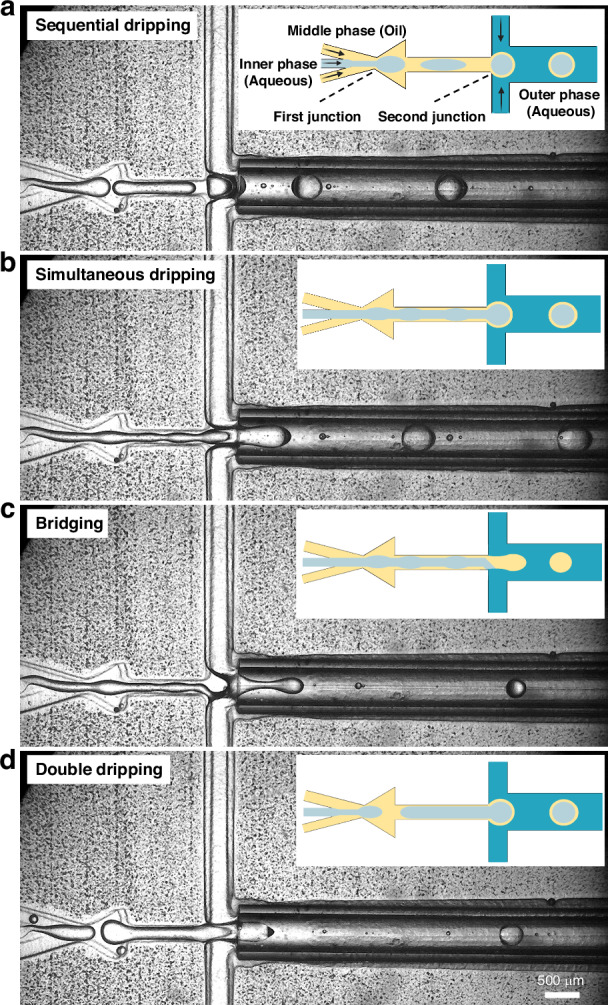
. Experimental and corresponding schematics representing different modes of thin shell double emulsion formation. In schematic the different phases are represented by different colors. Light blue (IP), yellow (MP) and dark blue (OP). **a** The sequential dripping can yield to the formation of thick or thin shell DES depending on the size of the inner emulsion. **b** Simultaneous dripping forms thin shell double emulsions. The transition from sequential dripping to simultaneous dripping can be achieved by increasing the IP pressure while maintaining the OP pressure constant. **c** Simultaneous dripping is highly unstable and prone to disruptions which may cause the coalescence of inner and outer phase streams, called bridging. **d** Double dripping mode used for the formation of thin shell double emulsions prevents bridging issue. Regarding pressures, double dripping requires an IP pressure level between the level in sequential and simultaneous dripping while having a higher OP pressure. The same chip design and capillary dimensions explained in Fig. 3c were used for these experiments

DEs must have a thin shell to serve as templates for microfluidic liposome formation^8,15,46^. Furthermore, generating ultra-thin shell DEs has been proposed to reduce solvent residue and improve double emulsion stability, hence expanding their potential applications^9,47^. Consistent production of such structures requires a specific formation mode which we study in detail (Video S3†). The first mode, sequential dripping (Fig. 5a), involves creating a W/O droplet at the first junction and then encapsulating it in the outer aqueous phase. The resulting double emulsions can be thick or thin shell, determined by the relative size of the W/O droplet to the final DE. While offering flexibility in shell control, this method has two main disadvantages: (i) It does not favor ultra-thin shell DE formation. (ii) It requires a change in the chip design to generate DEs smaller than IP channel dimensions at the first junction. As a second mode (Fig. 5b), simultaneous dripping is presented which is a more effective approach to achieve thin shell double emulsions. This method involves having a jetting regime for the Inner phase (IP) by increasing its Weber number^48^ and then dripping both the Inner IP and Middle phase (MP) at the second junction simultaneously. However, instabilities arise due to the high inertia of the IP stream and the high interfacial tension between the MP and the other two phases. These factors can lead to “bridging” (Fig. 5c), where IP and OP coalesce, resulting in oil-in-water (O/W) emulsions.

The final mode introduced is double dripping (Fig. 5d), where large W/O droplets are formed in the first junction and then dripped again alongside oil to form DEs in the second junction. This mode successfully mitigates bridging due to the reduced influence of inner phase inertia with formation of large W/O droplets while achieving a DE shell thickness comparable to simultaneous dripping. However, there is a risk of oil droplet formation in this mode^7,32,47^. This issue is addressed in the present study by developing a high-throughput separation method, explained in the following section.

Another aspect regarding the significance of formation modes is their response to the inlet pressure change as illustrated in Fig. S3†. In sequential dripping, reducing the outer phase pressure (P_OP_) leads to the formation of multicompartment double emulsions. Conversely, in the simultaneous dripping mode, a decrease in P_OP_ results in larger single-core double emulsions with minimal changes in the relative shell thickness. Therefore, one should select the most suitable formation mode based on their specific application.

### Sample purification by separation of double emulsions from oil droplets

**Fig. 6:**
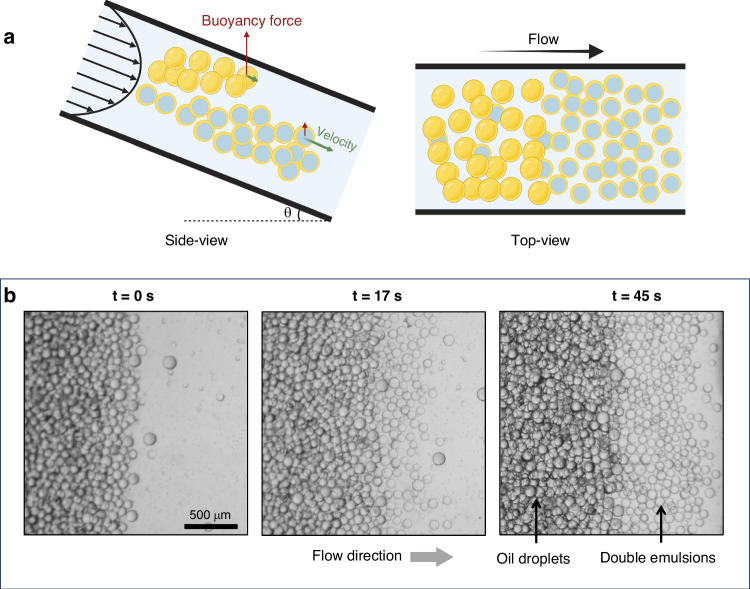
. Illustration of the presented method for separating double emulsions from oil droplets. **a** Schematic illustration of the separation process. The side-view and top-view sketches represent the position of DE and oil droplet with respect to channel walls. Higher buoyancy force in oil droplets leads to their proximity to the top wall, causing a lower velocity compared to DEs. **b** The velocity difference between DEs and oil droplets is shown by comparing the three snapshots from one experimental video. At t = 0 s both oil droplets and double emulsions are mixed. After t = 17 s since the double emulsions have higher velocity, they have started to separate from oil droplets. At t = 45 s the difference in distance and separation are more apparent

**Fig. 7:**
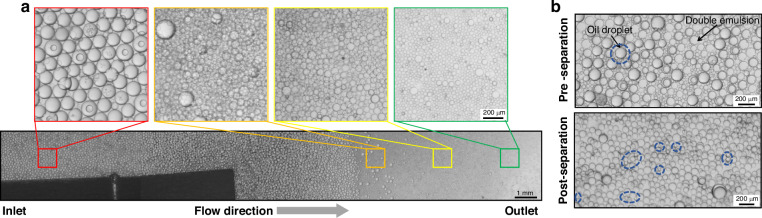
. The successful separation of double emulsions from oil droplets in the separation chip. **a** Oil droplets accumulate close to the inlet of the channel. Toward the outlet, the number of the oil droplets is significantly reduced, and double emulsions are separated. The insets are zoomed-in images from different sections of the separation chip. **b** A comparison between the sample before (pre-separation) and after the separation chip (post-separation) illustrates the effectiveness of our separation method. The blue dashed circles and ellipses are marking oil droplets (single or multiple) which appear to be more 3D-like and darker than DEs in the bright field images

The purity of the double emulsion (DE) sample is crucial not only for the formation of microfluidic liposomes but also for any subsequent physical or biological investigation. Oil droplet impurities in the DE sample alter the concentration of lipid solvent within the medium, wet the sample container, and merge with DEs. These issues hinder the transition from DE templates to liposomes and make further studies such as microanalysis impractical. Therefore, it is imperative that oil droplet contamination is removed during the DE^32^ or liposome preparation process^31,32^. To address this issue, we propose a rapid, high throughput and continuous method for isolating double emulsions from oil droplets. This approach involves utilizing a combination of fluid flow and density difference to achieve DE separation. As illustrated in Fig. 1a, a sealed chamber with a height of 1 mm is positioned at 25° inclined angle as a separation chip (See supporting information Part-III, Figs. S4† and S5† for the detailed discussion about the effect of inclination angle). The outlet of the hybrid device is connected to the separation chip to achieve continuous separation. By exploiting the inherent density differences between oil droplets and double emulsions, the design achieves distinct spatial distribution based on buoyancy force and fluid flow velocity (Fig. 6a). Oil droplets, being less dense than DEs, experience a stronger buoyancy force, propelling them upstream and towards the top wall of the chamber, where the flow velocity is minimal due to the parabolic velocity profile. Conversely, DEs, being denser and smaller, reside predominantly near the centerline, where the flow velocity is higher. This velocity disparity between double emulsions and oil droplets translates to spatial separation as they advance downstream (Fig. 6b, video S4†), ultimately leading to a highly purified sample at the separation chip outlet (Fig. 7, video S5†). For high-volume manufacturing of DEs, the separated oil droplets should be removed, which can be achieved by incorporating an additional inlet to flush the separation chip without stopping the formation (Fig. S6†). To quantify the performance of the separation chip, two metrics were used: separation efficiency (SE, Eq. 1) and enrichment factor (EF, Eq. 2)^49^. Figure 7b illustrates the effectiveness of the separation chip in purifying the sample yielding a 98% SE and 1.21 EF. It should be noted that the achieved 1.21 EF value is very close to the theoretical maximum of 1.23 (See supporting information Part-III for the calculations). Figure S7† illustrates the successful separation of DEs and oil droplets of similar size, indicating that the effectiveness of the separation process does not rely on the oil droplets being larger than the DEs.

1 $${\rm{SE}}={\left(\frac{{\rm{Number}}\; {\rm{of}}\; {\rm{DEs}}}{{\rm{Total}}\; {\rm{number}}\; {\rm{of}}\; {\rm{emulsions}}}\right)}_{{\rm{post}}-{\rm{separation}}}\times 100 {\text{\%}}$$

2 $${\rm{EF}}=\frac{{\left(\frac{{\rm{Number\; of\; DEs}}}{{\rm{Total\; number\; of\; emulsions}}}\right)}_{{\rm{post}}-{\rm{separation}}}}{{\left(\frac{{\rm{Number\; of\; DEs}}}{{\rm{Total\; number\; of\; emulsions}}}\right)}_{{\rm{pre}}-{\rm{separation}}}}$$

The proposed method stands out in comparison to off-chip droplet separation examples^31,47,50^, due to two key advantages. Firstly, it eliminates the need for double emulsion being denser than the outer phase, a constraint imposed by the off-chip separation method. This overcomes a significant limitation, expanding the applicability of our technique to a wider range of densities, given that double emulsions typically have a lower density than the outer phase. Secondly, the on-chip separation capability enables real-time purification and direct transfer of isolated double emulsions to subsequent microfluidic processes, a feature absent in off-chip approaches which offers minimizing sample handling and cross contamination.

### Assembly of giant liposomes from double emulsion template

**Fig. 8:**
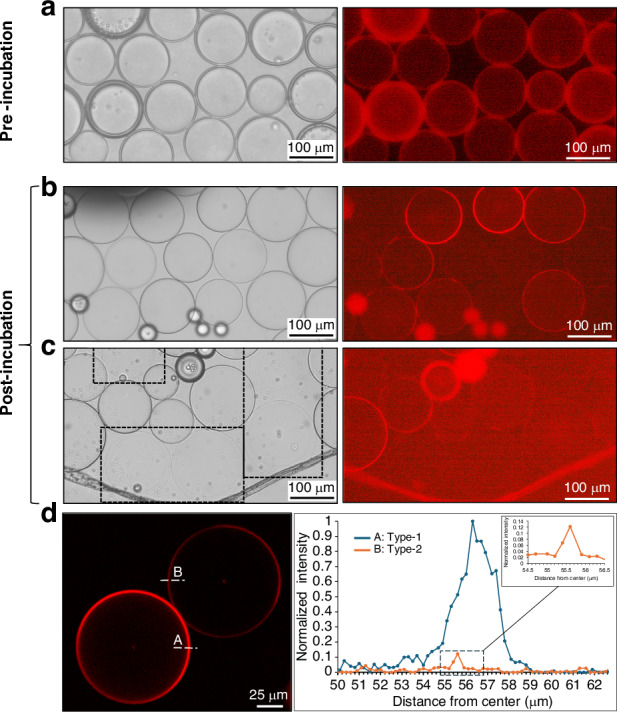
. Giant liposomes formation from double emulsion templates by solvent extraction method. **a** The bright field and fluorescence microscopy images from double emulsions formed using hybrid device after the transfer to the observation chamber. The shell thickness of double emulsions varies between 2.5 to 5 µm. Bright field and its corresponding fluorescent images from two distinct categories of liposomes formed after 15 h of incubation. **b** The first type of liposomes with the approximate thickness of 1 µm and (**c**) The second type of liposomes with a thickness of less than 400 nm which are marked by dashed rectangles in the bright field image. The remaining of the liposomes belong to the first type. **d** The confocal image of liposomes and its corresponding membrane fluorescent intensity profile from the first type (A) and second type (B), representing the significant difference between these two classifications. The membrane is stained by Liss Rhod PE

The hybrid device, as a versatile platform for generating double emulsions, specifically with the capability to generate thin shell double emulsions, emerges as a valuable tool for producing liposomes. Liposome formation by solvent removal from double emulsion templates is the most developed method for the microfluidic formation of cell-sized liposomes^3,4,6^. To validate the applicability of the hybrid device for giant liposome generation, the solvent extraction method was employed^32,33^. DEs were successfully formed using a combination of DOPC and cholesterol as phospholipids in the middle phase. The DEs were then transferred to the observation chamber and incubated for 15 h. During this period, the formation of liposomes from double emulsions involved a solvent-mediated extraction of excess oil. Ethanol, present in the outer phase, acted as a solvent to dissolve oleic acid, present in the middle phase, leading to the progressive reduction of oil content. The initial shell thickness of double emulsions ranged from 2.5 to 5 µm (Fig. 8a). Following the incubation period; based on the bright field microscopy images, two visually distinct types of liposomes emerged. The first type shown in Fig. 8b exhibited a shell thickness of approximately 1 µm. Despite having solvent traces in the membrane, these vesicles are suitable for applications involving bioreactions or cargo encapsulation. The second type shown in Fig. 8c displayed a shell thickness of less than 400 nm without any distinct boundary in the bright field image, suggesting higher membrane purity. Therefore, the outcome of this type can be considered a more reliable synthetic model for cell membrane research. The confocal image and its corresponding fluorescent intensity profiles clarify the difference between the shell thickness of these two types (Fig. 8d). Based on our results, the second type was observed less frequently. Achieving such high purity in the membrane is rare in the former solvent extraction studies^32–35^. We hypothesize that local solvent saturation caused this categorization. Liposomes in the first type likely formed in regions with high double emulsion density, facilitating faster ethanol saturation. Conversely, the second type emerged in areas with fewer double emulsions and higher outer phase interaction.

Achieving a high liposome generation yield is challenging in the solvent extraction method. Therefore, we elaborate on several key factors influencing liposome yield in this method: concentration of solvent, double emulsion shell thickness, lipid composition, and the size of double emulsion. Solvent concentration (ethanol) plays a crucial role in determining the outcome of the extraction process. Low concentrations of ethanol do not lead to liposome formation, whereas high concentrations cause rapid dissolution and destabilization of double emulsions^35^. Similarly, DEs with thick shells (typically more than 25% relative shell thickness) do not transform into liposomes following solvent extraction, while ultra-thin shell DEs (typically less than 5% relative shell thickness) do not retain stability which might be due to insufficient phospholipids to cover the membrane leaflets^32^. The experiments conducted by using cholesterol in the lipid composition provided higher stability of double emulsions and liposome formation yield indicating the importance of lipid composition. Additionally, we noticed that the portion of double emulsions that maintain their stability during the solvent extraction process in a sample with small-sized double emulsions (typically less than 150 µm size) was significantly higher than in a sample with large-sized double emulsions (typically more than 150 µm size).

## Conclusions

In this work, we presented a new microfluidic setup for liposome generation featuring treatment-free double emulsion formation, separation, and solvent extraction. It has been demonstrated that combining PDMS chip and glass capillary as the hybrid device provides a facile DE formation chip eliminating the need for surface treatment to form DEs and yields excellent results. This approach offers several additional advantages, including reusability, design flexibility and low fabrication complexity, addressing critical challenges associated with conventional DE generation methods. The versatile nature of the hybrid device is showcased in its ability to generate diverse double emulsions, allowing for variations in size (ranging from 27 µm to 1.2 mm), shell thickness, the number of compartments, and solvent choices. In addition, it has been illustrated that among the three modes of forming thin shell DEs, double dripping is the most robust mode for consistent formation. Furthermore, we have introduced a high throughput, continuous separation method using a modular chip, which can be integrated with the hybrid device to isolate double emulsions while excluding unwanted oil droplets. The outcome is a rapid, traceable, and on-chip density-based separation method. Ultimately, the solvent extraction process was utilized to convert double emulsion templates into giant liposomes. The results were categorized into two types distinguished by the amount of solvent residue content, thereby defining their respective potential applications. The presented microfluidic system lays a foundation for repeatable and controllable generation of liposomes and complex liposomal structures by providing an easy-to-implement and reliable method for double emulsion formation and separation.

## Experimental section

### Materials

Oleic acid (90%), 1-octanol (anhydrous, ≥99%), mineral oil (light oil), Polyvinyl alcohol (PVA, M_W_ = 13k–23k gmol^−1^, 87–89% hydrolyzed), glycerol ( ≥ 99% (GC)), cholesterol (3β-Hydroxy-5-cholestene) and 1,2-dioleoyl-sn-glycero-3-phosphoethanolamine-N-(lissamine rhodamine B sulfonyl) (Liss Rhod PE, ammonium salt) were purchased from Sigma Aldrich. Polyethylene glycol (PEG, powder/flakes, M_W_ = 6000 gmol^−1^ was purchased from Alfa Aesar. Poloxamer (P188, 10% solution) was obtained from Thermo Fisher. 1,2-di-(9Z-octadecenoyl)-sn-glycero-3-phosphocholine (DOPC) was purchased from Avanti Polar Lipids. Ethanol (absolute ≥99.8%) was purchased from VWR chemicals.

### Hybrid device fabrication

The hybrid device includes a PDMS chip and a glass capillary. The PDMS chip was fabricated using standard soft lithography. The PDMS chip’s mould was fabricated using stereolithography (SLA) 3D printing (3D printer Form 3 + , Formlabs, Gray resin, 25 μm resolution). Two sets of moulds were designed for two different sizes of glass capillaries. The mould intended for large capillary (1.4 mm outer diameter (OD) and 1 mm inner diameter (ID), QuikRead® 20 µl capillaries, Aidian Oy) has inlet channels measuring 100, 200 and 250 μm in width and 50, 100 and 125 μm in height, respectively, for IP, MP, and OP. Additionally, the outlet channel has a semi-circle cross-section with 1.4 mm diameter to accommodate the large glass capillary. The mould designed for the small capillary (820 μm OD and 550 μm ID, Microcaps® 6.66 μl 72 mm, Drummond Scientific) comprises inlet channels with widths of 50, 200 and 250 μm, and heights of 25, 100, 125 μm respectively for IP, MP and OP. Further, an outlet channel having a semi-circle cross-section of 800 μm diameter was designed to accommodate the small glass capillary. The utilization of 3D-printed moulds enables the implementation of a 3D-flow-focusing design. This results in the continuous phase enveloping the dispersed phase on all sides. Additionally, employing 3-D printing to fabricate the mould offers the distinct advantage of having a semi-cylindrical outlet channel, that provides seamless integration of the glass capillary within the chip.

To fabricate the PDMS chip replica, a 10:1 mixture of PDMS prepolymer and curing agent (Sylgard 184™ Silicone Elastomer Kit, Dow Corning) was cured in an oven at 80 °C for 2 h. Two identical PDMS replicas were fabricated from the same mould and surface activated in an oxygen plasma device (Colibri, Gambetti). Subsequently, the two replicas were aligned under a stereo microscope (SMZ-2B, Nikon) and bonded together. The final PDMS chip consists of inlet channels with a square cross-section and an outlet channel with a circular cross-section. The chip was then placed on a hot plate at 80 °C for 2 h.

The desired glass capillary is inserted into the outlet channel of the PDMS chip (Fig. 2b), ensuring that its tip is located in the second junction (Fig. 2d). To generate smaller double emulsions, the glass capillary was tapered with a microcapillary puller (PC-10 Puller, Narishige International) and then sanded to achieve the desired tip diameter.

### Double emulsion formation

For double emulsion formation demonstrating the versatility of the hybrid device (Figs. 3–5), the IP was an aqueous solution with 8 wt% PEG, the MP was oleic acid and the OP was an aqueous solution with 10 wt% PVA and 1 wt% P188. To show the flexibility in using different lipid solvents (Fig. 4c), in addition to oleic acid two other organic solvents, namely 1-octanol and mineral oil were used in MP. For the separation process (Figs. 6, 7), an aqueous solution with 8 wt% PEG as IP, oleic acid as MP and an aqueous solution with 5 wt% glycerol and 1 wt% P188 as OP were utilized. For liposome formation (Fig. 8), IP was a 1:1 mixture of P188 and deionized (DI) water. The OP was an aqueous solution containing 2.5% v/v P188, 14% v/v glycerol and 14% v/v ethanol, while the MP composed of 2 mgml^-1^ DOPC, 0.4 mgml^−1^ cholesterol and 3 µgml^−1^ Liss Rhod PE (For liposome membrane visualization). To prepare the MP for liposome formation, the required amount of each lipid stock solution (containing lipids dissolved in chloroform) was added to an amber glass vial. Chloroform was then evaporated from the solution using a vacuum dryer. Finally, the required amount of oleic acid was added to the dry lipid film in the vial and then sonicated for 30 min in a room-temperature bath.

The solutions corresponding to IP, MP, and OP were introduced into the hybrid device by using a pressure pump (OB1 MK3, Elveflow). The quality of treatment-free double emulsion formation is influenced by the solutions utilized in each phase. PEG was used in the inner phase, PVA and P188 in the outer phase to enhance the emulsification and stability of double emulsions. The size, number of compartments and shell thickness of the double emulsion were controlled by varying the inlet pressures at each phase. Due to the high frequency of the DE formation, the process was monitored by using an inverted optical microscope (DM IL LED, Leica) equipped with a high-speed camera (Fastcam Mini UX50, Photron). The monodispersity of double emulsions was analysed using DMV software^51^ and compared based on the CV index^52^. After generation, the double emulsions were transferred from the glass capillary to the separation chip via Tygon tubing.

### On-chip separation

The separation chip was fabricated by cutting the frame-shaped geometry out of a double-sided tape (1 mm thickness), using a laser cutter (Beambox Pro, Flux), which was sandwiched between a glass slide and a 3 mm PMMA sheet (Perspex® XT, 3 A Composites Group). The chip is placed in an inclined position at an angle of roughly 25 degrees, and connected to the hybrid device via Tygon tubing, thereby, coupling the separation chip with the hybrid device (Fig. 1a). Oil droplets and DEs forming in the hybrid device are transferred to the separation chip. The fluid flow from the hybrid device moves the DEs toward the outlet while oil droplets are captured within the separation chip. DEs are transferred from the outlet to the observation chamber via tubing. Notably, a sufficiently high flow rate (500 μl/min in our case) is crucial to generate a substantial velocity difference between DEs and oil droplets facilitating optimal separation. The separation process was monitored using a stereo microscope (MZ75, Leica) equipped with a CMOS camera (Flexacam c5, Leica). ImageJ software was used for counting emulsions to calculate separation metrics.

### Liposome formation

After formation, DEs were transferred to the observation chamber and incubated at room temperature for over 15 h. Two coverslips were used to fabricate the observation chamber using 1 mm thick double-sided tape as a spacer. Following the transfer we sealed the chamber using Vaseline (Vaseline® Blue Seal). The bright field and fluorescent images were collected using an inverted fluorescence microscope (DM IL LED, Leica) equipped with a CMOS camera (Flexacam c1, Leica). Confocal images were acquired using a confocal laser scanning microscope (TCS SP8 with a DMI8 microscope, Leica) and LASX 3.5.2 acquisition software was used to attain fluorescent intensity profiles.

## Supplementary information

Supplementary Information

video S1

Video S2

Video S3

Video S4

Video S5
