# Supplementary material for: Facile and versatile PDMS-glass capillary double emulsion formation device coupled with rapid purification toward microfluidic giant liposome generation
Source: Microsyst Nanoeng. 2024 Dec 5;10:183. doi: 10.1038/s41378-024-00815-0 (PMC11618511; doi:10.1038/s41378-024-00815-0)
Supplement: Supplementary file 1 — Supplementary Information [file 41378_2024_815_MOESM1_ESM.docx]

**Supporting Information for**

**Facile and versatile PDMS-glass capillary double emulsion formation device coupled with rapid purification toward microfluidic giant liposome generation**

**Mostafa Bakouei, ^1^ Ali Kalantarifard, ^1^ Indraja Sundara Raju, ^1^ Tatiana Avsievich, ^1^ Lauri Rannaste, ^1,2^ Marjut Kreivi ^2^ and Caglar Elbuken *^1,2^**

***^1^****Faculty of Biochemistry and Molecular Medicine, University of Oulu, FI-90014 Oulu, Finland*

***^2^****VTT Technical Research Centre of Finland, FI-90590 Oulu, Finland*

**Corresponding author*

**List of contents**

Part I. Supplemental details related to size tunability in double emulsion formation

Part II. Supplemental details related to double emulsion formation modes

Part III. Supplemental details related to sample purification by separation of double emulsions from oil droplets

Part IV. Supplementary videos

**Part I. Supplemental details related to size tunability in double emulsion formation**

**-Droplet size distribution analysis**


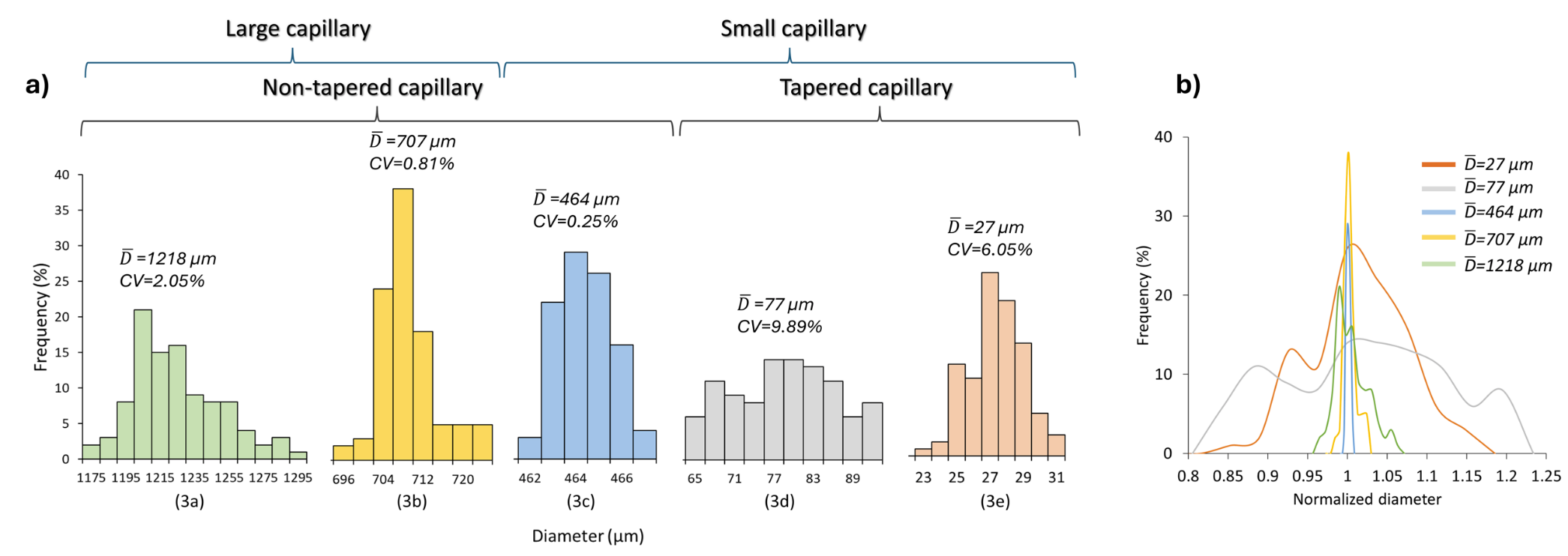


**Fig. S1 Characterization of monodispersity of resultant double emulsions (DE) using coefficient of variation (CV) index.**^1^ a) The size distribution of DEs corresponding to Fig. 3a-e. The monodispersity of resultant double emulsions was measured using n ≥ 100 DEs for each plot. DMV software was utilized to analyze the data.^2^ Ultra-monodispersity was achieved for the formation of 707 and 464 µm DEs. b) Normalized size distribution plot for generated DEs shown in Fig. 3, based on the average DE diameter. The coefficient of variation achieved in this study, ranging from 0.25% to 10%, is consistent with the typical range found in microfluidic liposome studies, which spans from 2% to 11%.^3–6^

**-Formation throughput, flow rates, and recommended capillary ID**

**Table. S1 Double emulsion formation throughput and flow rates correspond to different capillaries shown in Fig. 3.** High-throughput formation is achieved for thin shell DEs with less than 100 µm diameter. The throughput achieved in this work is on par with that reported in other microfluidic liposome generation studies for similar DE size.^3–5^

| Fig.3 | | a | b | c | d | e |
| --- | --- | --- | --- | --- | --- | --- |
| **Throughput** | | **1.3 DE/s** | **6 DE/s** | **10 DE/s** | **850 DE/s** | **1140 DE/s** |
| **Avg DE diameter (µm)** | | **1218** | **707** | **464** | **77** | **27** |
| **Capillary tip ID (µm)** | | **1000** | **1000** | **550** | **150** | **50** |
| **Flow rates (µl/min)**  *** (µl/hr)** | **I.P.** | 41 | 6 | 1.5 | 3 | 12.5* |
|  | **M.P.** | 22 | 17 | 7 | 5 | 17.5* |
|  | **O.P.** | 42 | 403 | 62 | 580 | 546* |

**Table. S2 recommended glass capillary tip inner diameter (ID)** for the formation of thin shell DE with different size ranges.

| DE size range (µm) | 20-50 | 50-200 | 200-600 | 600-1200 |
| --- | --- | --- | --- | --- |
| **Recommended capillary tip ID (µm)** | 50 | 150 | 550 | 1000 |

**-Shell thickness uniformity analysis**

**
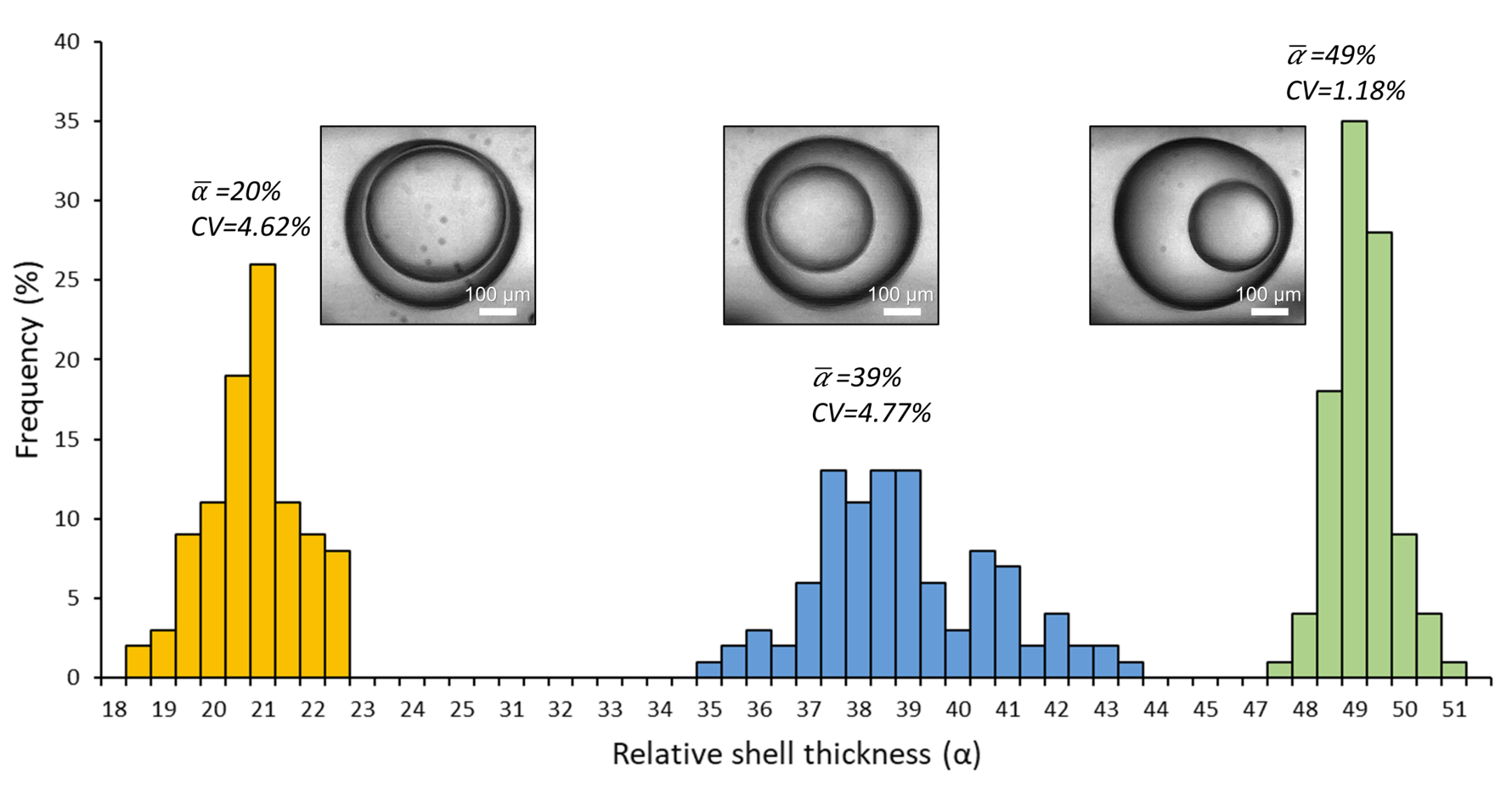
**

**Fig. S2 Characterization of shell thickness uniformity for DEs in Fig. 4a.** The shell thickness uniformity of DEs corresponding to Fig. 4a were analyzed and quantified using the CV index (n ≥ 100 DEs for each condition). The inset pictures represent DEs with corresponding shell thicknesses.

**Part II. Supplemental details related to double emulsion formation modes**

**
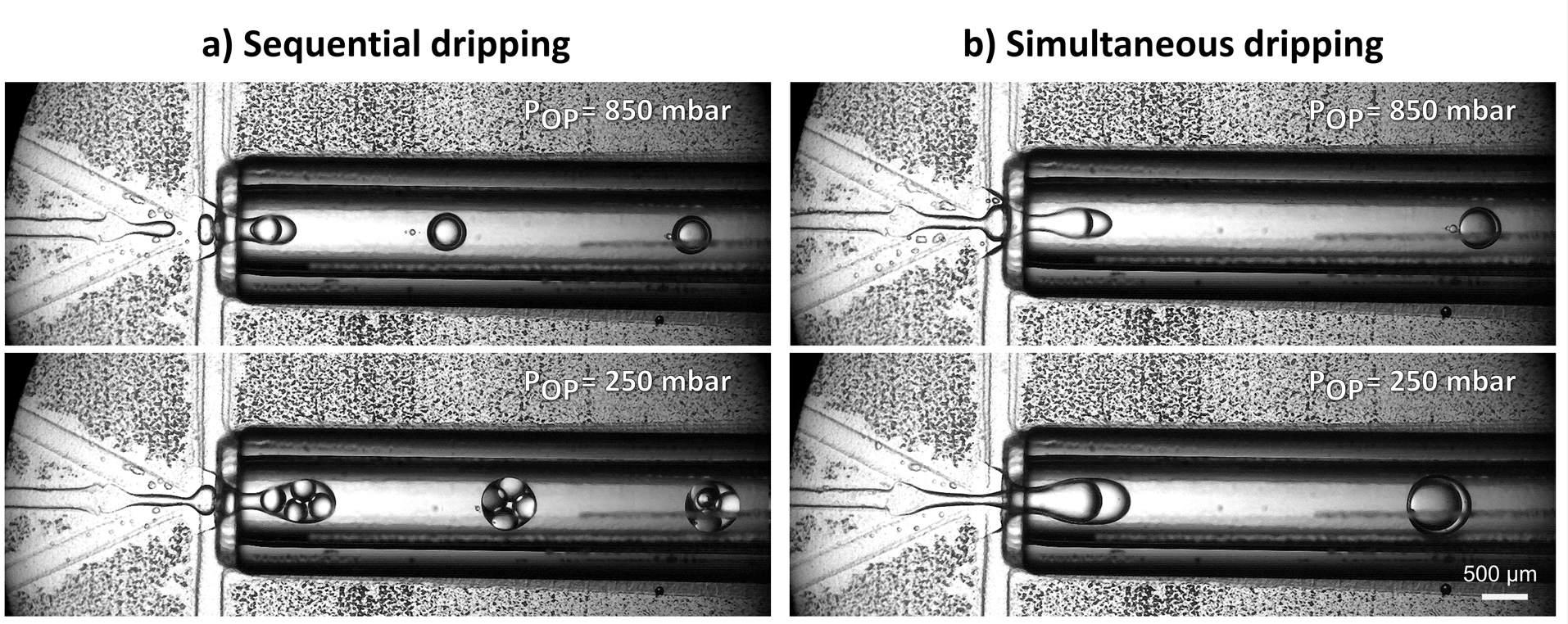
**

**Fig. S3 Formation mode’s response to the inlet pressure change.** Comparing the outcomes of sequential and simultaneous dripping modes in response to regulation of outer phase (OP) pressure. The reduction in OP pressure while maintaining other pressures the same yields: a) the formation of multicompartment emulsions and b) larger single-core double emulsions. The IP pressure was set to a) 40 mbar for sequential dripping and b) 70 mbar for simultaneous dripping while the MP pressure was set to 50 mbar for both modes. The chip design and capillary dimensions were the same as in Fig. 3a.

**Part III. Supplemental details related to sample purification by separation of double emulsions from oil droplets**

**-Discussion about the effect of inclination angle on the separation process:**

After testing different inclination angles, the recommended range for successful and high-throughput separation is between 15° to 35°. Here we compare the two extreme inclination angles with the 25° angle which was used for the obtained results. When no inclination is applied (i.e., horizontal chip), separation yields very low throughput. The absence of a force driving oil droplets upstream causes their downstream velocity to be higher than observed in 25° inclination. Consequently, this results in a reduced velocity disparity (Fig. S4) leading to fewer DEs being separated from the oil droplet region (Fig. S5). On the other hand, at a high inclination angle (vertical chip), several disadvantages arise: i) The buoyancy force does not drive oil droplets toward the top wall, thereby the parabolic velocity profile is not leveraged to establish a velocity disparity between DEs and oil droplets (Fig. S4). ii) Live imaging of the process becomes complicated using conventional microscopy systems by the need for side-imaging. iii) Gravitational flow caused by hydrostatic pressure renders the process less controllable.


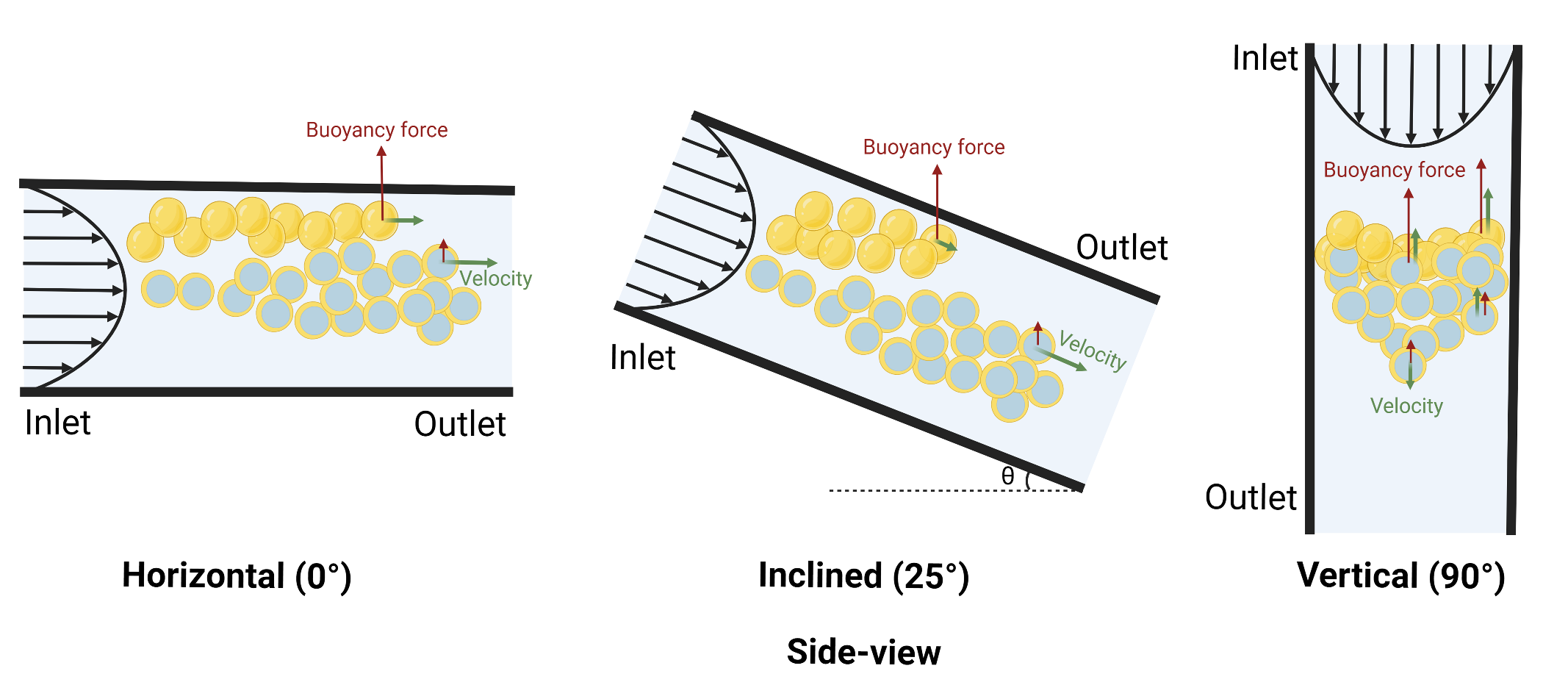


**Fig. S4 Schematic illustration of the effect of inclination angle on separation performance.** No inclination results in lower separation throughput due to reduced downstream velocity disparity between DEs and oil droplets compared to 25° inclination angle. A vertical chip does not favor separation, as it does not benefit from the parabolic velocity profile to create velocity disparity.

*
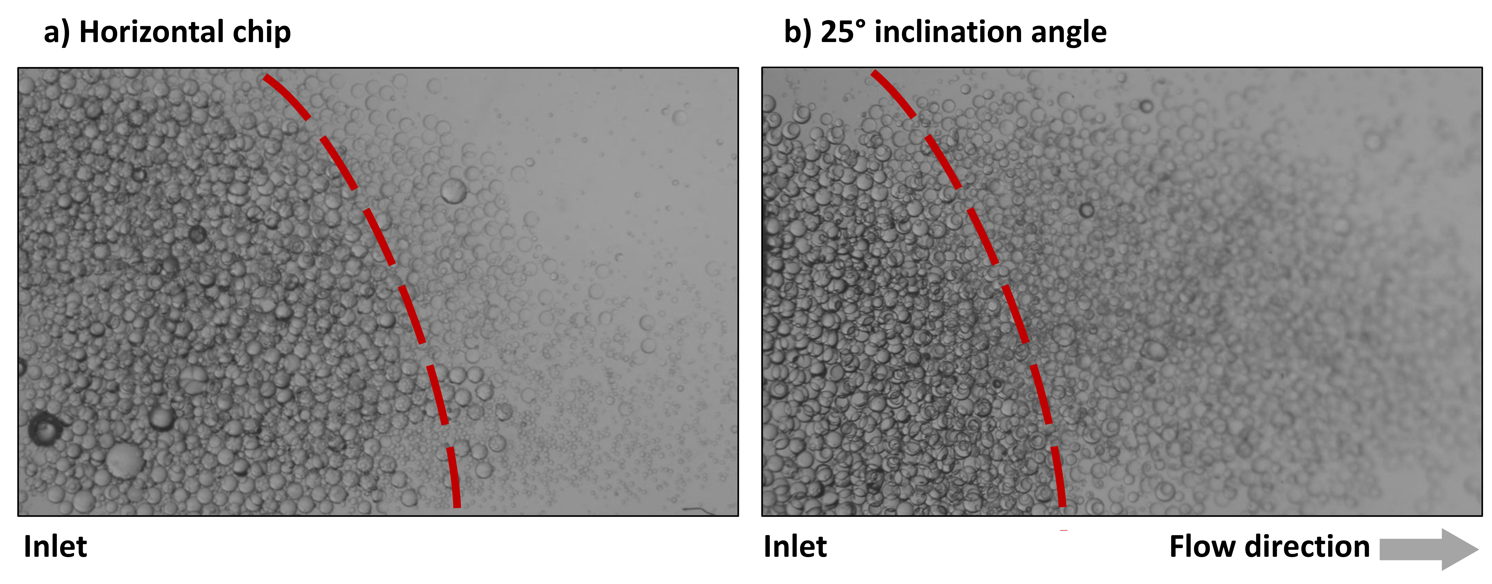
*

**Fig. S5 The effect of inclination angle on separation performance.** Both images were captured 30 seconds after starting the emulsion transfer to the separation chip. The oil droplet region (on the left side of the dashed line) is larger in the horizontal chip, resulting in fewer DEs being separated compared to the chip with a 25° inclination angle.

**
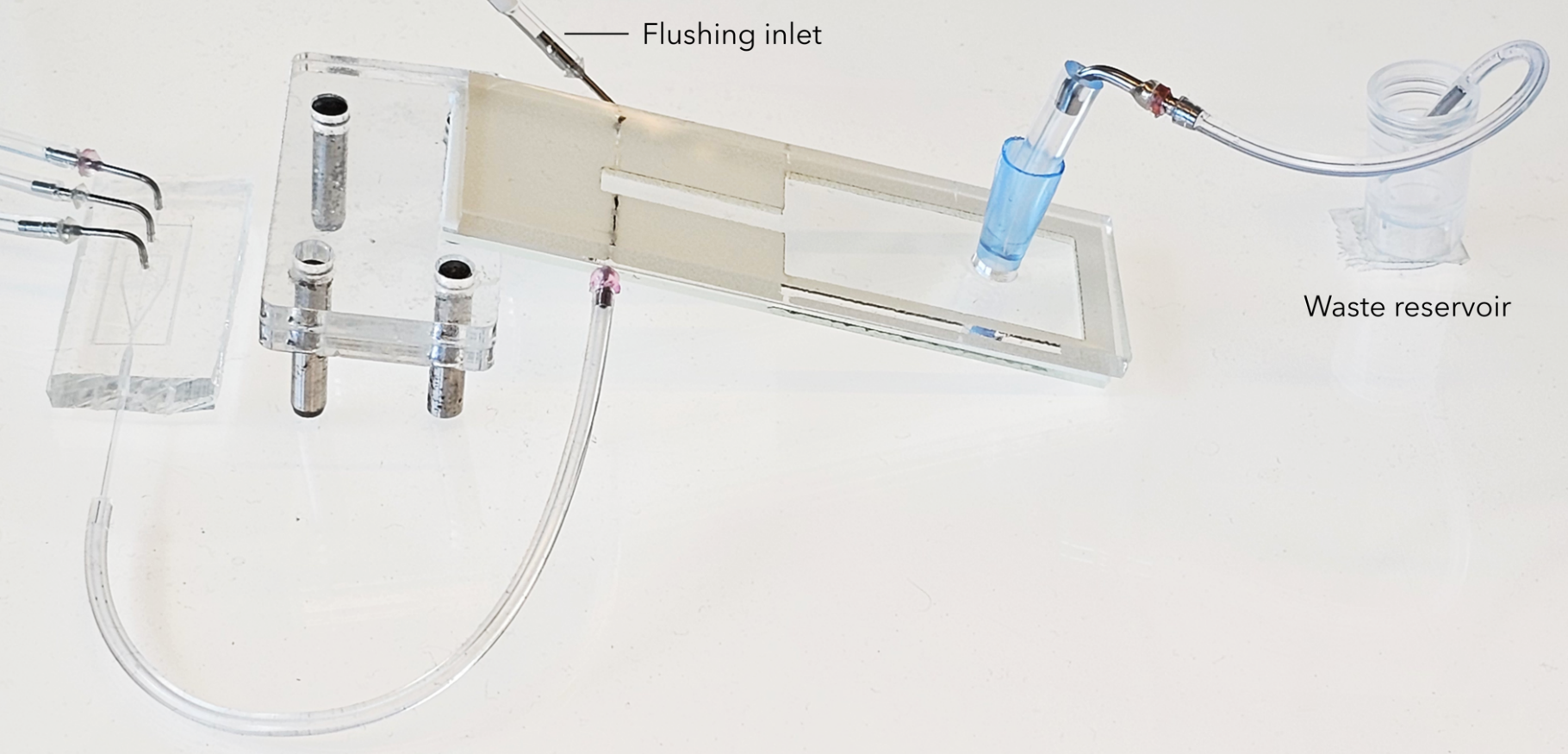
**

**Fig. S6 Flushing the separation chip for high-volume DE separation.** When the separation chip is accumulated with the leftover oil droplets, the outlet is connected to a waste reservoir, and the outer phase medium is perfused via flushing inlet to empty the separation chip. Therefore, the same chip can be used for the separation of new batches of DEs without stopping the formation process.

*
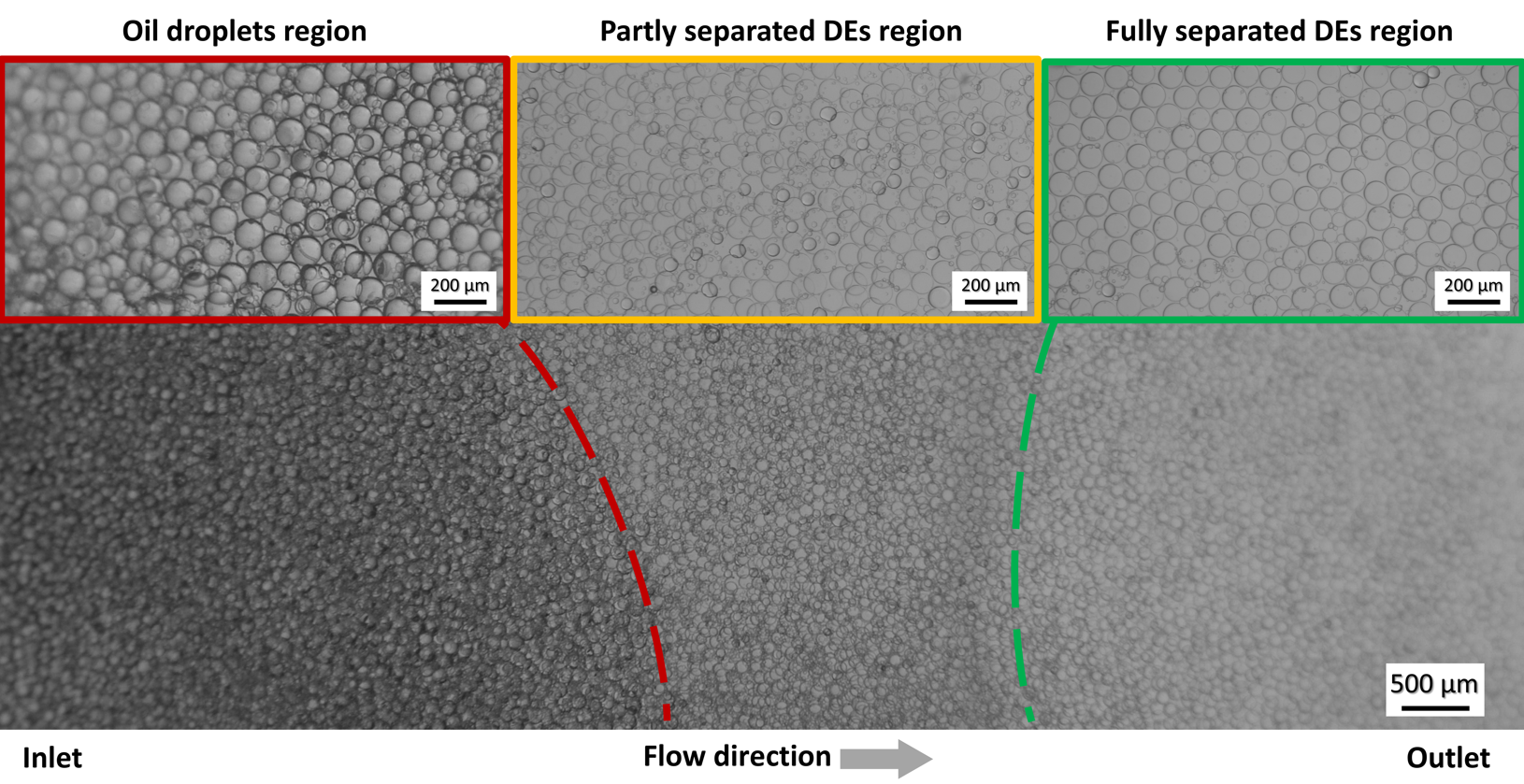
*

**Fig. S7 Separation of DEs from oil droplets in the separation chip.** A demonstration of successful separation of 100 µm DEs from similar-sized oil droplets using the proposed separation method. This result shows that the separation process succeeds even if DEs and oil droplets are in the same size range.

**-Calculation of separation efficiency and enrichment factor**

To calculate the separation efficiency (SE), a sample image including 651 emulsions was analyzed in which 641 of them were DEs and 10 were oil droplets resulting in 98% SE.

To calculate the enrichment factor (EF) the above numbers were used for post-separation. For pre-separation, a sample image including 961 emulsions was analyzed of which 783 were DEs and 178 were oil droplets. Considering these numbers the EF is 1.21 being very close to the theoretical maximum of 1.23.

EF = $\frac{\text{(}{\frac{\text{Number of DEs}}{\text{Total number of emulsions}}\text{) }}_{\text{post-separation}}}{\text{(}{\frac{\text{Number of DEs}}{\text{Total number of emulsions}}\text{) }}_{\text{pre-separation}}}=\frac{\frac{\text{641}}{\text{651}}}{\frac{\text{783}}{\text{961}}}\text{=}\frac{\text{0.98}}{\text{0.81}}\text{=}\text{ }\text{1.21}$

Max theoretical EF $\text{=}\frac{\frac{\text{651}}{\text{651}}}{\frac{\text{783}}{\text{961}}}\text{=}\frac{\text{1}}{\text{0.81}}\text{=}\text{ }\text{1.23}$

**Part IV. Supplementary videos**

**Video S1.** Tunability of double emulsions size using the hybrid device. All the double emulsion formation videos were recorded using a high-speed camera at different frame rates per second (FPS). The playback frame rate for all videos is 20 FPS.

**Video S2.** Formation of ultra-thin shell double emulsion using the hybrid device

**Video S3.** Thin shell double emulsion formation modes

**Video S4.** Separation of double emulsions from oil droplets caused by their velocity disparity

**Video S5.** High-throughput separation of double emulsions from oil droplets using the presented purification approach

**References:**

1. Kalantarifard, A., Alizadeh-Haghighi, E. & Elbuken, C. A microfluidic droplet system for ultra-monodisperse droplet generation: A universal approach, *Chem. Eng. Sci.* **261**, 117947 (2022).

2. Basu, A. S. Droplet morphometry and velocimetry (DMV): A video processing software for time-resolved, label-free tracking of droplet parameters. *Lab Chip* **13**, 1892–1901 (2013).

3. Deshpande, S., Caspi, Y., Meijering, A. E. C. & Dekker, C. Octanol-assisted liposome assembly on chip. *Nat Commun* **7**, 10447 (2016).

4. Deng, N. N., Yelleswarapu, M. & Huck, W. T. S. Monodisperse Uni- and Multicompartment Liposomes. *J Am Chem Soc* **138**, 7584–7591 (2016).

5. Chien, P. J., Shih, Y. L., Cheng, C. T. & Tu, H. L. Chip assisted formation of phase-separated liposomes for reconstituting spatial protein-lipid interactions. *Lab Chip* **13**, 2540-2548 (2022)

6. Michelon, M., Huang, Y., de la Torre, L. G., Weitz, D. A. & Cunha, R. L. Single-step microfluidic production of W/O/W double emulsions as templates for Β-carotene-loaded giant liposomes formation. *Chemical Engineering Journal* **366**, 27–32 (2019).
